# Lupus Nephritis Associated With Renal Cell Carcinoma

**DOI:** 10.7759/cureus.31870

**Published:** 2022-11-24

**Authors:** Vivek Mehta, Darpan Gandhi

**Affiliations:** 1 Rheumatology, Alaska Native Tribal Health Consortium, Anchorage, USA; 2 Nephrology, Alaska Native Medical Center, Anchorage, USA

**Keywords:** male lupus, rheumatology & autoimmune diseases, carcinomas renal cell, sle and lupus nephritis, lupus nephritis

## Abstract

A 43-year-old man was found to have hematuria and proteinuria during a regular checkup. Workup revealed a renal mass on a CT scan. He continued to have worsening proteinuria and hematuria, with declining renal function, so a renal biopsy was performed, which revealed lupus nephritis class IV. He underwent partial nephrectomy, which confirmed the diagnosis of renal cell carcinoma on pathology. He was started on glucocorticoids, hydroxychloroquine, and cyclophosphamide for lupus nephritis.

## Introduction

Systemic lupus erythematosus (SLE) is an uncommon autoimmune condition that can affect almost any organ of the body. It is more commonly seen in young women but can also be seen in men. When SLE presents in men, it tends to have a more severe presentation with worse long-term outcomes [1]. Lupus nephritis (LN) can be seen in up to 50% of patients with SLE, often resulting in end-stage renal disease despite immunomodulatory therapies [1,2].

Renal cell cancer (RCC) originates from the renal epithelium and accounts for >90% of all cancers in the kidney, with incidence increasing with advancing age [3]. RCC accounts for 2% of all cancers and is noted to be more common in men [4].

## Case presentation

A 43-year-old man was found to have microscopic hematuria and proteinuria on work-related medical clearance. A month later, he underwent cystoscopy, which was unrevealing, so a CT scan of the abdomen and pelvis was performed, revealing a 1.5 cm mass concerning for renal cell carcinoma (RCC). 

**Figure 1:**
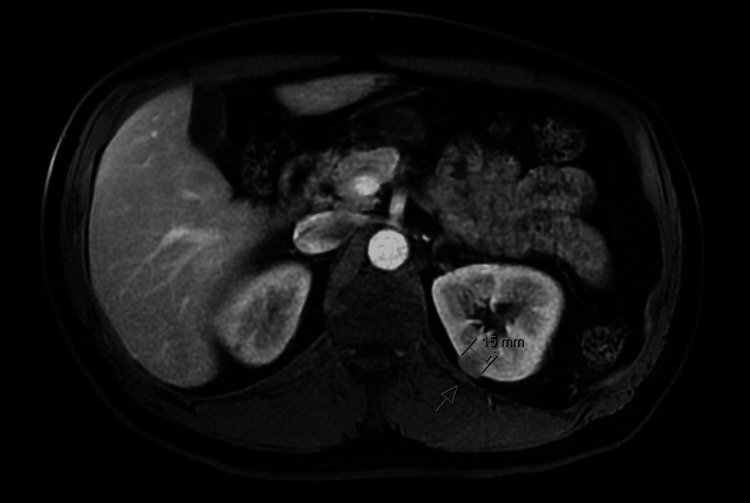
MRI of the abdomen showing renal mass (arrow)

The patient's initial presentation with hematuria and proteinuria was attributed to possible RCC, which was visualized on a CT scan. The mass was further characterized with an MRI of the abdomen (Figure 1).

Further workup was pursued when his protein/creatinine ratio worsened from 4.7 to 8.5. He was unable to provide a 24-hour urine sample so proteinuria was followed by protein/creatinine ratios. He was found to be negative for anti-nuclear antibody (ANA) but surprisingly had elevated double-stranded DNA antibody (dsDNA Ab) at 12 IU/mL (normal range 0-9). He was noted to have low C3 (10 mg/dL, normal range 14-44 mg/dL) and C4 (57 mg/dL, normal range 82-167 mg/dL), CH50 was normal at >60 units/ml (normal value >41 units/mL). His anti-neutrophilic cytoplasmic antibodies (ANCAs) were negative. His other workup, including antibodies to extractable antigens, Sjogren antibodies, scleroderma antibodies, and anti-phospholipid antibodies, was negative. His creatinine had increased up to 5.5 mg/dL (normal range 0.6-1.3 mg/dL), and glomerular filtration rate (GFR) decreased to 11 mL/min at the time of diagnosis; creatinine has stabilized at 2 mg/dL and GFR at 35 mL/min after treatment. 

**Figure 2:**
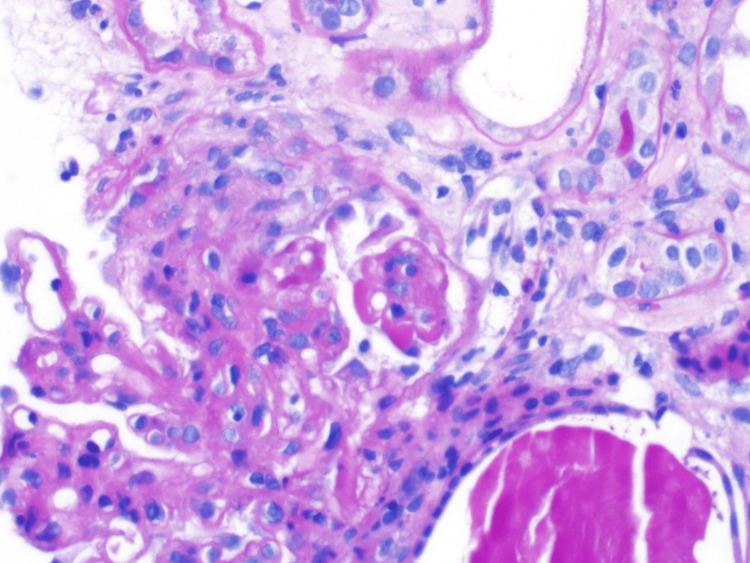
Kidney biopsy pathology with findings of lupus nephritis

He underwent a renal biopsy due to worsening renal function. Pathological findings were consistent with diffuse lupus nephritis (LN) class IV with an activity score of 13/24 and a chronicity score of 3/12 (Figure 2).

The decision was made to proceed with partial nephrectomy for renal mass before initiating potentially carcinogenic immunomodulatory treatment for LN class IV. Tissue examination from partial nephrectomy revealed chromophobe renal cell carcinoma with a clear margin.

In consultation with the patient's urologist, nephrologist, and rheumatologist, the decision was made to proceed with partial nephrectomy. A 2.5 cm mass with clear margins was excised, and pathology confirmed the diagnosis of RCC. He was started on cyclophosphamide 500 mg IV every two weeks per Euro-Lupus protocol for lupus nephritis, prednisone, and hydroxychloroquine, which led to a partial response. Following cyclophosphamide Euro-Lupus protocol for three months, he was switched to Mycophenolate Mofetil with further improvement in proteinuria. 

## Discussion

SLE is an autoimmune disorder that often affects multiple organs. People with SLE are shown to have a higher malignancy risk. The most common malignancy in SLE is non-Hodgkin's lymphoma (NHL); a higher risk of vulva, lung, thyroid, breast, and possibly liver malignancy has also been noted in prior studies [5,6]. People with SLE often require immunosuppressive medications, which also predisposes them to various malignancies [7].

It is unusual to have SLE with negative ANA; many previously reported negative SLE cases were thought to be due to sera were checked with older nonspecific testing methods [8]. Many ANA-negative patients have also been reported to have Anti-SSA/Ro antibodies, which were negative in this patient [9]. C2 deficiency can lead to SLE with negative ANA; this was ruled out by normal CH50 levels [10]. Proteinuria leading to immunoglobulin loss, prozone effect, and longer disease duration are some of the other possible etiologies of ANA-negative SLE reported in literature [11].

Chromophobe renal cell carcinoma is a rare subtype of RCC, seen in about 5% of RCC patients and often presenting in earlier stages [12]. It can be seen with a familial form of RCC Birt-Hogg-Dubé syndrome that was not suspected in his case, so no genetic testing was pursued. This patient has been monitored with serial imaging without any signs of recurrence.

This patient had an unusual presentation of hematuria and proteinuria, which were initially attributed to RCC suspected on imaging. However, when his hematuria and proteinuria progressed, a kidney biopsy was pursued, which revealed the findings of lupus nephritis. There are limited reports of SLE patients presenting with RCC. Goupil et al. reported a case of LN presenting with renal mass who did not have underlying malignancy, concluding that it would be helpful to keep this in differential when evaluating a patient of SLE with a renal mass [13]. Matsuda et al. reported a case of an LN patient who had undergone treatment with steroids and cyclophosphamide, who was later to be found to have RCC [14]. Wong reported a patient with LN who was found to have RCC due to persistent hematuria despite completing treatment with steroids, cyclophosphamide, and Mycophenolate. It was postulated that the RCC was likely present before or at the time of diagnosis of LN [15]. Gopalkrishnan et al. reported a case of SLE who had been in remission with prednisone and azathioprine for eight years, found to have RCC when she underwent ultrasound for evaluation of pyelonephritis [16].

Our patient was found to have RCC and LN concurrently. Malignancies have been shown to increase autoimmunity, and autoimmune disorders have been shown to have increased malignancy risk [5,6,7,17]. An atypical presentation of SLE or malignancy should alert one to the possibility of this complex overlap, which can help with early diagnosis.

## Conclusions

SLE is an uncommon autoimmune disorder with increased malignancy risk. Atypical presentation due to malignancy and SLE overlap can occur. SLE manifestations like lupus nephritis carry the risk of high mortality and morbidity; early identification is essential so that prompt treatment can be started. When the clinical picture is conflicting, imaging and biopsy can provide useful data for further management. SLE patients should be monitored closely for concurrent malignancies; early diagnosis can lead to improved clinical outcomes. 
